# New surgical realities: implementation of an enhanced recovery after surgery protocol for gynecological laparoscopy—a prospective study

**DOI:** 10.1186/s13741-021-00221-4

**Published:** 2021-12-15

**Authors:** Jose Carlos Vilches Jimenez, Beatriz Tripiana Serrano, Emilia Villegas Muñoz, Belinda Sanchez Pérez, Jesús S. Jimenez Lopez

**Affiliations:** 1 Department of Obstetrics and Gynecology, Hospital Regional Universitario Malaga, Av. del Arroyo de los Ángeles, s/n, 29011 Malaga, Spain; 2 Department of General Surgery, Hospital Regional Universitario Malaga, Av. de Carlos Haya, 84, 29010 Malaga, Spain

**Keywords:** ERAS protocol; Gynecology, Laparoscopic hysterectomy, Benign disease, Cohort study

## Abstract

**Background:**

Multimodal rehabilitation allows optimization of functional recovery in surgery patients by reducing the postoperative stress and hospital stay duration, without increasing the morbidity and mortality. It is reportedly successful in other surgical disciplines, and guidelines for its application to gynecological surgery are available; however, most evidence for these guidelines is derived from observational and/or retrospective studies. Therefore, this study aimed to investigate the applicability of an enhanced recovery after surgery (ERAS) protocol in laparoscopic gynecological surgery and its influence on the postoperative stay, morbidity, mortality, and readmission, through a prospective approach.

**Methods:**

This prospective cohort study was performed on 90 patients who underwent laparoscopic hysterectomy for benign causes from October 2017 to October 2019. Patients in whom the ERAS (ERAS group, *n* = 30) and traditional (control group, *n* = 60) protocols were implemented were compared. All patients were followed for 6 months.

**Results:**

The groups were homogeneous and did not differ significantly with respect to the demographic characteristics (age, ASA score, body mass index), surgical indications, and surgery types. Adherence to the ERAS protocol was over 99%. The postoperative hospital-stay durations were 1.73 days (*r* = 1–3) and 2.97 days (*r* = 2–6) in the ERAS and control groups, respectively (*p* = 0.000). No significant intergroup differences were observed in the rates of complications and readmissions.

**Conclusions:**

The ERAS protocol is applicable in laparoscopic gynecological surgery and can be implemented with good adherence. This can allow optimization of patient recovery by reducing the hospital stay duration, without increasing the rates of morbidity, mortality, or readmission.

## Introduction

The practice of multimodal surgical rehabilitation, also known as the intensive recovery program, “fast-track surgery,” or “enhanced recovery after surgery” (ERAS), first emerged at the end of the twentieth century with works by Kehlet (Kehlet, 1997) and Bardram et al. (Bardram et al., 1995). Although ERAS or fast-track surgery protocols were first adopted for colorectal surgeries, the good outcomes obtained have allowed their extension to other specialties.

These programs have a multidisciplinary approach, and their main objective is to improve patient recovery after surgery. To achieve this, patient involvement is fundamental, and it is necessary to develop a pathway that addresses the preoperative assessment and optimization, patient nutrition, surgical analgesia, the surgery itself, and postoperative management.

Currently, there are reference guidelines for the implementation of these protocols within gynecological surgery (Nelson et al., 2016a; Nelson et al., 2016b). However, even with the last update of 2019 (Nelson et al., 2019), part of the evidence for the application of different techniques and procedures is derived from studies in other surgical specialties. Another problem is that many of the studies carried out in gynecology are retrospective and/or observational (Nelson et al., 2019; Nicholson et al., 2014).

The paradigm shift with this protocol is substantial and has provoked criticism and rejection. Despite having demonstrated a clear improvement in perioperative outcomes, these protocols suffered from a massive lack of adherence in their early days and still suffer in many places today. This fact is quite striking, and even Kehlet himself has attempted to address it in his 2017 publication *Enhanced Recovery After Surgery: Current Controversies and Concerns* (Kehlet & Joshi, 2017).

Therefore, this study aimed to assess the implementation of an ERAS protocol in patients undergoing laparoscopic hysterectomy in a level III center.

The main objective of this study was to achieve the implementation of an ERAS protocol in laparoscopic gynecological surgery. The secondary objectives were to assess its influence on the postoperative stay and the morbidity, mortality, and readmission rates and to assess patients’ adherence to ERAS protocol too.

## Material and methods

### Patients

This prospective cohort study enrolled 90 patients who presented to the Hospital Regional Universitario de Málaga from October 2017 to October 2019. Patients who were scheduled for a laparoscopic hysterectomy for benign causes were included, while those who required conversion to open surgery, resided outside our province, and whose ASA score was > II were excluded.

These patients were categorized into two groups: (1) the ERAS group (*n* = 30, patients in whom the ERAS protocol was implemented) and (2) the control group (*n* = 60, patients in whom the traditional protocol was implemented). All patients were followed for 6 months. The main differences between the two protocols can be seen in Table 1.

**Table 1:** Main differences between the protocols

ERAS (n30)	Traditional protocol (n60)
Pre-surgical optimization	No pre-surgical optimization
Carbohydrate-rich diet the day before surgery	Normal diet
6-h fast for solids and 2-h fast for clear liquid	8 h fast for solids and liquids
Maintain euvolemia during surgery	No euvolemia during surgery
Active heating during surgery	No active heating during surgery
Laparoscopic port infiltration	No laparoscopic port infiltration
Restrictive fluid therapy after surgery.	Prolonged fluid therapy
Tolerance 6 h after surgery	Tolerance 1 day after surgery
Avoiding the use of opiates	Opiates are allowed
Removal of bladder catheter 12–24 h after surgery	Removal of bladder catheter 24–48 h after surgery
Active mobilization 1st day PO	Active mobilization 2nd day PO

### Sample size

The sample size was calculated based on the sample size calculation for the comparison of two means (Charan & Biswas, 2013). We estimated the minimum sample size necessary to obtain a decrease in hospital stay of at least 1 day in the ERAS group compared to the traditional protocol. The mean length of stay during the year prior to the study for laparoscopic total hysterectomy was 3.2 days and for subtotal hysterectomy 3.13 days. Furthermore, this difference was estimated for a risk *α* of 5% and a power of 90%. Under these conditions, the minimum group size was 17.13 patients.

Finally, it was decided to perform a 1:2 study, and 30 patients were included in the ERAS group and 60 in the control group. The patients were divided into groups according to the ABBABB scheme.

### Pre-surgical visit

Pre-surgical visits are one of the pillars of our protocol. During this visit, patients were informed of the procedure to be performed, and the procedure itself was optimized for surgery. Pre-surgical visits were always managed by a gynecologist from our surgery unit.

### Surgery

The surgeries themselves were performed by the gynecological surgery unit of our hospital and were attended by at least one senior surgeon. Furthermore, all personnel involved in the process, i.e., the surgeons, anesthesiologists, and nurses, received specific training on the phases and procedures defined in the ERAS protocols before the study start. Likewise, our center’s protocol was also developed by adapting the evidence gathered in the available clinical guidelines (Table 2) (Nelson et al., 2016a; Nelson et al., 2016b; Nelson et al., 2019; Grupo GERM, protocolo histerectomía y miomectomia, n.d.).

**Table 2:** The ERAS protocol

**Preoperative**	
Inform the patient of her surgery and the protocol to be followed in the consultation	
Give up tobacco, alcohol, and ACHOs 4 weeks before surgery, correcting anemia	
Carbohydrate-rich diet the day before surgery	
6-h fast for solids and 2-h fast for clear liquid	
Abdominal and vaginal shaving (if necessary) of the patient	
No mechanical bowel preparation	
**Intraoperative**	
Anesthetic induction and anesthetic maintenance with short-acting agents	
Maintain temperature 36 °C ± 0.5 (thermal blankets, hot sera)	
Pneumatic compression stockings	
Antibiotic prophylaxis	
Fluid therapy in continuous perfusion balanced solution (3–5 ml/kg/h for laparoscopy), maintain euvolemia	
Hemodynamic optimization through objective-guided fluid therapy (FGO) in risk patients	
Postoperative nausea and vomiting prophylaxis with double therapy	
No drainage, no nasogastric tube	
Infiltration of the laparoscopy ports with bupivacaine	
**Immediate postoperative**	
Active temperature maintenance, maintenance of FiO_2_ 0.5 2 h after the end of the operation	
Analgesia according to the operation, minimum morphic administration, avoid opiods	
Restrictive fluid therapy.	
Start of oral tolerance at 6 h postsurgery, if positive oral tolerance, liquid diet in the evening and removal of intravenous fluids Beginning of mobilization and prophylaxis of the thromboembolism at 6 h after surgery	
**Postoperative**	
Blood test the morning after the intervention	
Normal balanced diet according to tolerance	
Removal of bladder catheter 12–24 h after surgery	
Active mobilization	
Oral analgesia according to protocol, avoiding morphs, breathing incentive	
Assess discharge from laparoscopic surgery (24–48 h)	

### Follow-up

After discharge, the patients underwent an in-person check-up 1 month after the surgery and were followed for up to 6 months to confirm a return to normal activity and to rule out any complications. All postoperative complications were classified according to the Clavien–Dindo classification (Clavien et al., 2009).

### Outcomes

#### Main outcome

- Achieve the implementation of an ERAS protocol in laparoscopic gynecological surgery

#### Secondary outcomes

- Assess ERAS protocol influence on the postoperative stay and the morbidity, mortality, and readmission rates
- Assess patients’ adherence to ERAS protocol

### Statistical analysis

All statistical analyses were performed using the SPSS package v25 (IBM). Qualitative variables were described by counts and frequencies, while quantitative variables were described by means, medians, and ranges. The patient characteristics and distribution were compared between the two groups by using Student’s *t* test and the *χ*^2^ test. The level of statistical significance was set at *p* = 0.05.

### Data collection and analysis

The entire multidisciplinary team involved in the protocol audited their part of the process.

- The principal investigator analyzed the data obtained.
- Nursing checks on admission the items that should have been carried out at home. In the operating theater, they check pre/intraoperative care.
- Anesthesiologists record their intraoperative and immediate postoperative performance.
- The gynecologists write up the clinical evolution of the patients on the ward until they are discharged. They also record the data of the preoperative and postoperative visits.
- Everything is reflected in the patient’s electronic history.

### Ethical approval

All the patients were informed and consented to the elaboration and publication of this research. Similarly, the ethics committee of our center gave its approval.

## Results

The total adherence to the designed protocol was higher than 99%, based on the evaluation of 14 items from the entire process (Table 3).

**Table 3:** Adherence to protocol

	Group ERAS
**Preoperative counseling**	100%
**6-h fasting**	100%
**Compression stockings**	100%
**Antibiotic prophylaxis**	100%
**Restrictive fluid therapy**	100%
**Nausea and vomiting prophylaxis**	100%
**Active heating**	100%
**Avoiding drainage**	100%
**NSAIDs as contributors**	100%
**Laparoscopic port infiltration**	100%
**Tolerance 6 h after surgery**	100%
**Removal of bladder catheter 1st day PO**	100%
**Avoiding the use of opiates**	96.67%
**Active mobilization 1st day PO**	96.67%
**Total adherence**	99.52%

As shown in Table 4, the ERAS and control groups were homogeneous with respect to the demographic characteristics (age, ASA, and body mass index), surgical indications, and type of surgery.

**Table 4:** Group characteristics

	ERAS (n30)	Traditional protocol (n60)	***p*** value
**Age (years)**			
Mean (± *SD*)	42.97 (± 7.88)	43.07 (± 9.51)	NS
Median (min/max)	45 (24/56)	44.5 (21/60)	
**ASA score**			NS
ASA I (%)	46.7	36.7	
ASA II (%)	53.3	63.3	
**BMI (kg/m2)**			
Mean (± *SD*)	25.83 (± 3.66)	26.60 (± 5.14)	NS
Median (min/max)	25 (20/34)	25 (17/39)	
**Surgical indication (%)**			NS
Symptomatic myoma	66.7	63.3	
Gender identity disorder	23.3	25	
Others	10	11.7	
**Surgical procedures (%)**			NS
Total hysterectomy	40	46.7	
Subtotal hysterectomy	60	53.3	

*Abbreviations*: *BMI* body mass index, *SD* standard deviation

The mean hospital stay duration was 1.73 days (*r* = 1–3) in the ERAS group and 2.97 days (*r* = 2–6) in the control group protocol (*p* = 0.000). Up to 40% of the patients in the ERAS group were discharged from the hospital on the first postoperative day.

The incidence of postoperative complications was 6% in the ERAS group and 20% in the control group; this difference was not statistically significant (*p* = 0.1). In the subgroup analysis of complications (Clavien–Dindo I, II, and III), no significant intergroup differences were observed as well. Furthermore, no grade IV complications were reported. The analysis and distribution of complications are shown in Table 5.

**Table 5:** Complications recorded in both groups

	ERAS (n30)	Traditional protocol (n60)	***p*** value
**Total**	2 (6%)	12 (20%)	NS
**Grade I**	1 (3%)	6 (10%)	NS
**Grade II**	1 (3%)	5 (8.33%)	NS
**Grade III**	0	1 (1.6 %) Vaginal cuff dehicence	NS
**Readmission in relation with postoperative complications**	0	1 (1.6 %) Vaginal cuff dehicence	NS

According to Clavien–Dindo classification

## Discussion

Our study showed that the implementation of an ERAS protocol in gynecological laparoscopic surgery is feasible and decreases the hospital stay duration, without increasing the rates of readmission and complications associated with the surgery. Even though there is no statistical significance regarding the reduction of complications, there does appear to be a tendency for fewer complications to occur in the ERAS group (6% vs 20% *p* = 0.1). This difference could reach statistical significance with a larger *n*.

Despite the fact that ERAS protocols are becoming the new standard for the management of gynecological surgery, much of the evidence and procedures are derived from protocols and studies performed in other surgical specialties (Nicholson et al., 2014). Moreover, studies comparing these protocols in gynecological surgery are usually observational in nature and/or compare the ERAS group with retrospective control cohorts (Minig et al., 2015; Lambaudie et al., 2017). As indicated by de Groot et al. (de Groot et al., 2016) in their review and meta-analysis of published studies, the main problem in gynecological surgery is the reliance on observational studies that carry a high risk of bias. Scheib et al. again emphasize in their subsequent literature review the need for gynecological own studies that proves the ERAS thesis and the importance of quantifying compliance in these studies (Scheib et al., 2019).

Although it is true that there are clinical trials that have attempted to validate these protocols, these are mainly focused on oncological surgery and their results are inconsistent. For instance, Dickson et al. (Dickson et al., 2017) found no significant differences in the hospital stay duration between the ERAS and traditional protocols, while Ferrari et al. (Ferrari et al., 2020) reported a shorter hospital stay duration for the ERAS protocol as compared to the standard protocol.

Within benign surgery, we found fewer studies, although benign hysterectomy is one of the main procedures within gynecology. Yilmaz et al. performed a clinical trial to evaluate abdominal hysterectomy with shortened length of stay (Yilmaz et al, 2018). Dr. Olga Kilpiö’s group presented a clinical trial to evaluate laparoscopic hysterectomy in the ERAS group but only evaluated length of hospital stay and use of opioids. Other elements of ERAS are not taken into account, and compliance is not assessed (Kilpiö et al, 2020).

There are two main difficulties associated with the implementation of the ERAS protocol. First, the need to coordinate and train multiple professionals from different specialties requires a great deal of collaboration from everyone involved in the process. It is essential to reach a protocol that is agreed upon and adapted to the reality of each center and that allows the traceability of each of its items. Reviewing compliance with these items and identifying possible failures or problems are fundamental to the implementation phase, because adherence is directly related to the success of the ERAS program, as shown by several studies (Gustafsson et al., 2011; Iniesta et al., 2019).

Second, our study population initially rejected the reduction in the hospital stay duration, believing that they could receive less attention than necessary for their procedure. Therefore, pre-surgical re-education of patients should be prioritized in order to resolve the doubts and fears that the new protocol may generate. It is also important to design a personalized follow-up calendar for the patients. In our case, all these visits were scheduled and managed by gynecologists during the implementation process; in some studies, the nursing staff has been trained to coordinate these actions on a larger scale and with a greater number of patients, once the implementation process has been completed. Furthermore, the follow-up visits were performed in person. With a view of expanding the scale of the program, the possibility of including telephone visits after the first 30 days following surgery has been considered in order to combine both actions, as recommended in the literature (Wong et al., 2014).

Our study has some limitations, including its sample size and the absence of a cost study. However, we believe that its strengths outweigh its weaknesses: it is a prospective study with homogeneous groups, a long-term follow-up, and an adherence-to-the-ERAS-protocol rate of above 99%.

## Conclusion

Our study shows that the implementation of an ERAS protocol, and the evidence developed in other surgical specialties regarding it, is also applicable in gynecological laparoscopic surgery. Therefore, it is a feasible option for optimizing the recovery of our patients and reducing their hospital stay, without increasing the rates of complications and readmissions.

## Data Availability

The datasets used and analyzed during the current study are available from the corresponding author on reasonable request (jcvilches@sego.es).

## Abbreviations

ERAS: Enhanced recovery after surgery
BMI: Body mass index
SD: Standard deviation
*r*: Range
PO: Postoperative
